# Ein 63-jähriger Patient mit akuter Sehverschlechterung nach perforierender Keratoplastik bei Keratokonus

**DOI:** 10.1007/s00347-020-01226-y

**Published:** 2020-09-15

**Authors:** Amine Maamri, Loïc Hamon, Loay Daas, Berthold Seitz

**Affiliations:** Klinik für Augenheilkunde, Universitätsklinikum des Saarlandes (UKS), Kirrberger Str. 100, Gebäude 22, 66421 Homburg/Saar, Deutschland

## Anamnese

Ein 63-jähriger Patient stellte sich zur Mitbeurteilung bei schwerer Hornhaut-Endothel-Epithel-Dekompensation am linken Auge nach externer perforierender Keratoplastik (PKP) bei Keratokonus im Jahr 1990 vor.

Der Patient klagte über eine schnell zunehmende und persistierende Sehverschlechterung seit 3 Wochen am linken Auge ohne Augenschmerzen. Außer den Augenoperationen (extern durchgeführte perforierende Keratoplastik und extern durchgeführte limbusparallele Keratotomien) waren die Allgemein- und die Familienanamnese unauffällig. Der Patient gab an, keine orale oder systemische Medikation einzunehmen.

## Klinischer Befund

**Figure 1:**
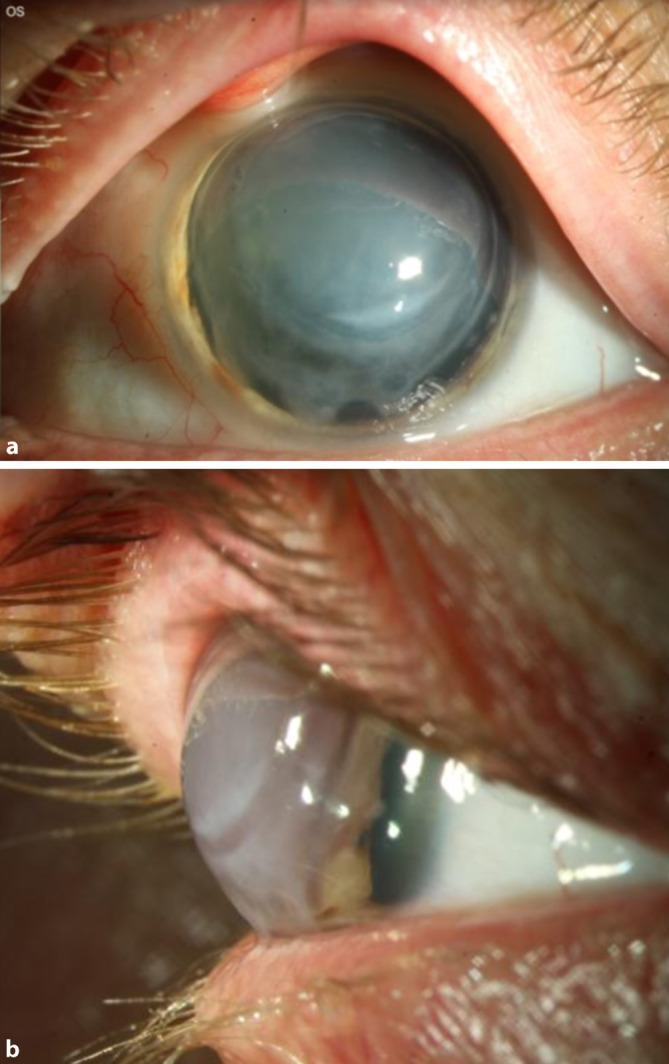

Bei der Erstvorstellung betrug der unkorrigierte Visus am betroffenen linken Auge Handbewegung. Gläser konnten diesen nicht bessern. Spaltlampenbiomikroskopisch zeigten sich am betroffenen linken Auge eine schwer dekompensierte Hornhaut sowie limbusparallele Keratotomien zur Korrektur eines hohen regulären Astigmatismus im Jahr 2002. Außerdem bestand eine große epitheliale Bulla von 7 bis 10 Uhr (Abb. 1).

Der fundoskopische Einblick war am linken Auge extrem reduziert. Sonographisch lag die Netzhaut allseits an.

## Diagnostik

**Figure 2:**
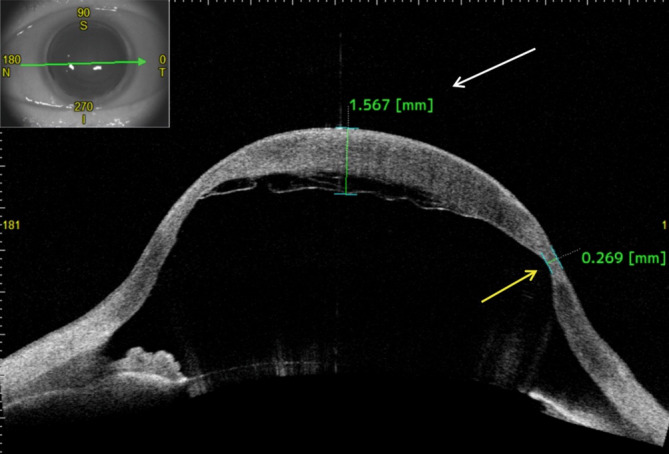

In der optischen Kohärenztomographie des vorderen Augenabschnitts (VA-OCT) beobachteten wir am linken Auge einen kornealen Hydrops aufgrund einer fokalen Ruptur der Descemet-Membran des Transplantats (Abb. 2 und 3).

**Figure 3:**
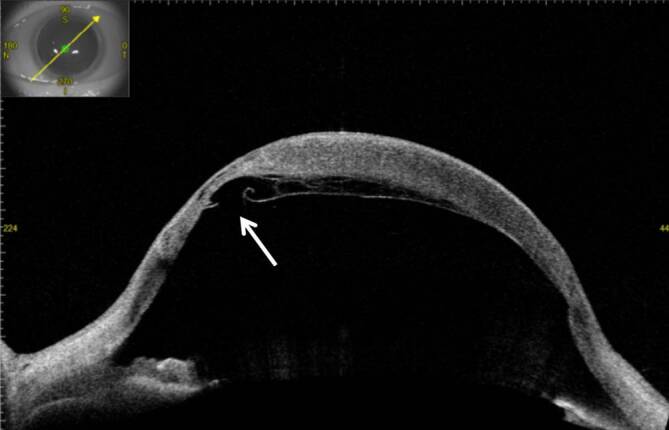

## Wie lautet Ihre Diagnose?

## Definition

Beim akuten kornealen Hydrops entwickelt sich ein ausgeprägtes stromales Hornhautödem aufgrund eines Risses der Descemet-Membran und wahrscheinlich der Dua-Schicht typischerweise bei Keratokonus (sog. „akuter Keratokonus“) [4, 7]. Zwei mögliche morphologische Aspekte eines akuten kornealen Hydrops wurden von Basu et al. beschrieben: Ablösung der Descemet-Membran mit Riss und eingerollten Enden (54 %) – so wie bei unserem Patienten – und Ablösung der Descemet-Membran mit Riss und flachen Enden (42 %) [2].

Die akute sekundäre Transplantatinsuffizienz durch kornealen Hydrops ist eine extrem seltene Komplikation bei Zustand nach perforierender Keratoplastik bei Keratokonus [4]. Diese wurde zum ersten Mal 1980 von Abelson et al. beschrieben [1] und tritt meistens 10 bis 28 Jahre nach der ersten perforierenden Keratoplastik auf [3]. In unserem Fall trat der akute korneale Hydrops 30 Jahre nach der ersten PKP auf.

Grundsätzliche Risikofaktoren für ein Transplantatversagen nach der PKP sind eine Hornhautvaskularisation, Aphakie oder Pseudophakie, Vorliegen von anterioren oder posterioren Synechien, lange Operationszeit und ein älteres Empfängeralter. Auch eine rezidivierende endotheliale Immunreaktion kann zum Transplantatversagen führen. Die Immunreaktion wird bekanntlich begünstigt durch eine Hornhautvaskularisation, lange Operationszeit, jüngeres Spenderalter [6] und v. a. einen großen Transplantatdurchmesser im Vergleich zur Größe der Wirtshornhaut [5].

## Therapie und Verlauf

Der Patient wurde am Tag der Untersuchung auf die Warteliste für eine perforierende Excimerlaser-Rekeratoplastik (geplante Größe [Empfänger/Spender]: 8,5/8,6 mm) gesetzt. Sowohl durch die Durchführung eines Excimerlaser-Schnitts als auch durch die große Dimensionierung des Transplantats kann ein postoperativer Astigmatismus deutlich reduziert werden [9, 10]. Die Ergebnisse einer Rekeratoplastik nach akuter sekundärer Transplantatinsuffizienz durch kornealen Hydrops sind insgesamt günstig und zeigen selten ein Rezidiv [2]. Je nach Fall ist es auch möglich, bei einem sekundären endothelialen Transplantatversagen nach perforierender Keratoplastik eine „Descemet membrane endothelial keratoplasty“ (DMEK) durchzuführen. Diese Behandlungsoption ist in Betracht zu ziehen, wenn die Hornhaut noch „durchsichtig“ ist (ohne stromale Narbe) und der Patient vor dem Hydrops mit seinem Visus zufrieden war [8]. Im Fall unseres Patienten war die Dekompensation zu schwerwiegend, und die Option einer DMEK stand nicht zur Debatte.

Wir leiteten eine lokale Therapie mit 5 % natriumchloridhaltigen Augentropfen zwecks einer Verlangsamung der Progression des Hydrops ein, solange der Patient auf der Warteliste für die PKP stand.

**Diagnose:** Akute sekundäre Transplantatinsuffizienz durch kornealen Hydrops

Fünf Wochen nach der Operationsindikation in unserer ambulanten Sprechstunde führten wir die geplante perforierende Excimerlaser-Keratoplastik am linken Auge in Intubationsnarkose durch. Das Transplantat wurde mittels 26 Einzelknüpfnähten (EKN) fixiert. Hierbei war es sehr schwierig, die extrem verdünnte Hornhaut Ende-zu-Ende an das Transplantat ohne eine „Minus-Stufe“ zu adaptieren. Intraoperativ bekam der Patient 2 g Ceftriaxon intravenös (i.v.), 250 mg Methylprednisolon i.v. und 500 mg Acetazolamid i.v.

Am nächsten postoperativen Tag zeigte sich der Seidel-Test negativ, das Transplantat war klar mit Descemet-Falten, die Vorderkammer stand, und 26 EKN waren fest.

Postoperativ erhielt der Patient am linken Auge als lokale Therapie Hyaluronsäure Augentropfen (AT) 5‑mal/Tag, Ofloxacin AT 5‑mal/Tag für 2 Wochen und Prednisolonacetat 1 % AT 5‑mal/Tag (mit Reduktionsschema von 1 AT alle 6 Wochen). Systemisch erhielt er Methylprednisolon per os 100 mg mit Reduktionsschema von 20 mg alle 2 Tage sowie Omeprazol 40 mg pro Tag. Nach 4 postoperativen Tagen konnte der Patient bei regelrechtem Befund entlassen werden. Kontrolltermine wurden für 6 Wochen, 9 Monate, 1 Jahr (mit Entfernung der 1. Hälfte von EKN) und 18 Monate (mit Entfernung der 2. Hälfte von EKN) vereinbart, zwischen welchen regelmäßige Kontrollen beim niedergelassenen Augenarzt geplant wurden.

**Figure 4:**
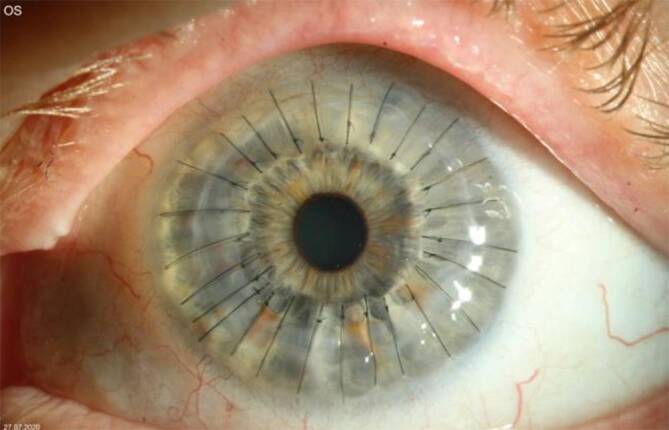

Sechs Wochen nach der Operation kam der Patient zur Kontrolle. Der Visus betrug am linken Auge sine correctione 0,4. Das Transplantat war klar. Es zeigten sich weder Abstoßungszeichen noch eine Erosio oder Fadenlockerung (Abb. 4 und 5).

**Figure 5:**
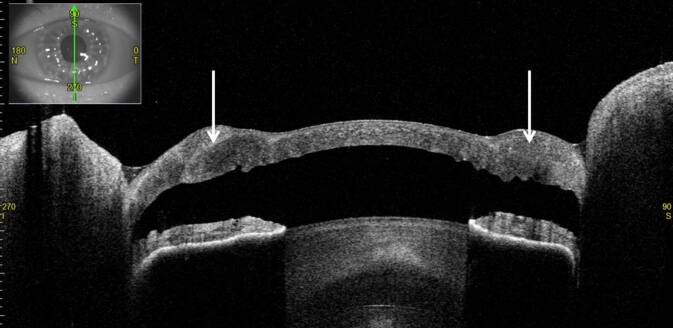

Eine eilige Rekeratoplastik ist im Fall einer schweren akuten sekundären Transplantatinsuffizienz durch kornealen Hydrops mit peripherer Stromaverdünnung die Therapie der ersten Wahl, da der Einblick für eine DMEK typischerweise nicht ausreichend ist. Hierbei ist die Excimerlaser-gestützte PKP die von uns favorisierte Methode, da mit dem konventionellen mechanischen Rundmesser bei der extrem verdünnten Wirtshornhaut weder eine runde Exzision noch perpendikuläre Kanten erzeugt werden können. Ursächlich kann eine Überdehnung der Descemet-Membran durch die starke Protrusion der Hornhaut aufgrund der zunehmenden Verdünnung im Bereich der Spender-Empfänger-Stoßstelle diskutiert werden. Im Entferntesten könnte auch an eine Mikroverletzung der Descemet-Membran bei den lange zuvor erfolgten limbusparallelen Keratotomien gedacht werden.

## Fazit für die Praxis

- Der korneale Hydrops des Transplantats stellt bei Keratokonus eine seltene Ursache für eine akute sekundäre Transplantatinsuffizienz dar. Trotzdem sollte der behandelnde Augenarzt immer daran denken.
- Das VA-OCT spielt hierbei die entscheidende Rolle für die Diagnose sowie die Einleitung der zielführenden Therapie.
- Eine eilige perforierende Excimerlaser-gestützte Rekeratoplastik ist im Fall einer schweren akuten sekundären Transplantatinsuffizienz durch kornealen Hydrops mit peripherer Stromaverdünnung die Therapie der ersten Wahl, da der Einblick für eine DMEK typischerweise nicht ausreichend ist.
